# Forgotten but not gone in rural South Africa: Urinary schistosomiasis and implications for chronic kidney disease screening in endemic countries

**DOI:** 10.12688/wellcomeopenres.18650.1

**Published:** 2023-02-10

**Authors:** Alison Craik, Mwawi Gondwe, Nokthula Mayindi, Shingirai Chipungu, Bongekile Khoza, Xavier Gómez-Olivé, Stephen Tollman, John Frean, Laurie A. Tomlinson, June Fabian

**Affiliations:** 1 Department of Non-Communicable Disease Epidemiology, Faculty of Epidemiology and Population Health, London School of Hygiene & Tropical Medicine, London, UK; 2 Medical Research Council/Wits University Rural Public Health and Health Transitions Research Unit (Agincourt), School of Public Health, Faculty of Health Sciences, University of the Witwatersrand, Johannesburg, South Africa; 3 International Network for the Demographic Evaluation of Populations and their Health (INDEPTH), Health and demographic surveillance systems, Accra, Ghana; 4 University of the Witwatersrand, Johannesburg, South Africa; 5 Centre for Emerging Zoonotic and Parasitic Diseases, National Institute for Communicable Diseases, National Health Laboratory Service, Johannesburg, South Africa; 6 Wits Donald Gordon Medical Centre, School of Clinical Medicine, Faculty of Health Sciences, University of Witwatersrand, Johannesburg, South Africa

**Keywords:** Schistosomiasis, chronic kidney disease, proteinuria, albuminuria, neglected tropical diseases, South Africa, sub-Saharan Africa

## Abstract

**Background:** Urinary schistosomiasis caused by *Schistosoma haematobium* ( *S. haematobium*) infection remains endemic in Africa and is associated with haematuria and albuminuria/proteinuria. The Kidney Disease Improving Global Outcomes clinical practice guidelines recommend evaluating proteinuria/albuminuria and glomerular filtration rate for chronic kidney disease (CKD) diagnosis. The guidelines are informed by population data outside of Africa but have been adopted in many African countries with little validation. Our study aimed to characterise the burden of urinary schistosomiasis in rural South Africa and evaluate its relationship with markers of kidney dysfunction with implications for CKD screening.

**Methods:** In this baseline population-based cohort study, we recruited 2021 adults aged 20–79 years in the Mpumalanga Province, South Africa. Sociodemographic and anthropometric data were recorded, urinalysis performed, and serum creatinine and urine albumin and creatinine measured. Kidney dysfunction was defined as an estimated glomerular filtration rate (eGFR) <60ml/min/1.73m ^2 ^and/or urine albumin-creatinine ratio >3.0mg/mmol. *S. haematobium* infection was determined by urine microscopy. Multivariable analyses were performed to determine relationships between *S. haematobium* and markers of CKD.

**Results:** Data were available for 1226 of 2021 participants; 717 (58.5%) were female and median age was 35 years (IQR 27 – 47). Prevalence of kidney dysfunction was 20.2% and prevalence of *S. haematobium* infection was 5.1%. *S. haematobium* infection was strongly associated with kidney dysfunction (OR 8.66; 95% CI 4.10 – 18.3) and related to albuminuria alone (OR 8.69; 95% CI 4.11 – 18.8), with no evidence of an association with eGFR <90ml/min/1.73m ^2^ (OR 0.43; 95% CI 0.05 – 3.59).

**Conclusions:** The strong association between urinary schistosomiasis and albuminuria requires careful consideration when screening for CKD. Screening for, and treating, schistosomiasis should be a routine part of initial work-up for CKD in *S. haematobium* endemic areas. Urinary schistosomiasis, a neglected tropical disease, remains a public health concern in the Mpumulanga province of South Africa.

## Introduction

Schistosomiasis persists in deprived communities, ranking low on national and global health agendas despite evidence that prevention and control are one of the “best buys” in global public health for neglected tropical diseases ^1^. Globally, an estimated 90% of schistosomiasis cases occur in Africa ^2^. In South Africa, 25 million people are at risk of infection, with 5 million (of which half are children) already infected ^3^. Endemic areas in South Africa report most infections due to *Schistosoma haematobium* ( *S. haematobium*) with the highest national prevalence in Limpopo and Mpumalanga provinces ^4^. Human infection occurs through direct contact with infected freshwater. Contributing factors for infection are pervasive water insecurity, increasing exposure to contaminated freshwater sources, and the absence of implemented programs for the monitoring, evaluation, preventive chemotherapy and vector management ^4^.

The lifecycle of *S. haematobium* begins with the shedding of eggs from the urine of infected hosts. Eggs hatch in optimal freshwater conditions, releasing miracidia which penetrate snails (intermediate host), and infected snails release larvae which penetrate human skin (definitive host) migrating through several tissues before lodging in the venous plexus of the bladder ^5^. Clinical manifestations depend on the host immune response, which may be cell- or immune-complex-mediated. A hallmark of the cell-mediated response is the “granuloma”, which can occur anywhere along the genitourinary tract and evolves from acute cellular inflammation (terminal hematuria, dysuria, frequency) to parenchymal-mesenchymal transformation dominated by fibrosis and calcification (may be asymptomatic). Fibrosis can result in strictures of the ureter, ureterovesical junction, or urethra, predisposing to urinary obstruction and reflux. Untreated chronic infection increases the risk of squamous cell carcinoma of the urinary tract. Renal disease may arise from chronic pyelonephritis or obstructive nephropathy ^6– 8^. There are a small number of case reports of *S. haematobium*-associated glomerular disease ^9– 11^.

As non-communicable disease (NCD) prevalence rises in Africa, the nexus between persistent infectious disease burdens (endemic, seasonal, and others) and NCDs is relevant and remains understudied ^12^. Data are scarce on the effect of *S. haematobium* infection on estimated glomerular filtration rate (eGFR), but its associations with leukocyturia, hematuria, and proteinuria/albuminuria are well documented, with some evidence of reversal following treatment with praziquantel ^6, 13, 14^. The Kidney Disease Improving Global Outcomes (KDIGO) guidelines for chronic kidney disease (CKD) diagnosis define CKD as (i) eGFR <60 ml/min/1.73 m ^2^, and/or (ii) markers of kidney damage - most commonly albuminuria and urine sediment abnormalities, that persist for at least three months ^15^. While the guidelines acknowledge a different context may require variations in practice, there is little guidance to inform CKD screening in schistosomiasis-endemic countries. Our study aimed to characterise the burden of urinary schistosomiasis in rural South Africa and evaluate how *S. haematobium* infection might impact markers of kidney damage used for diagnosing CKD.

## Methods

### Study design and participants

The African Research on Kidney Disease (ARK) Study aimed to determine CKD prevalence and identify associated risk factors in rural South Africa (SA) ^16^. The study took place from November 2017 to September 2018 and included a population-based sample (N=2759) of adults aged 20–79 years from the Agincourt Health and Socio-Demographic Surveillance System (HDSS) site in rural Bushbuckridge, Mpumalanga Province ^17^. Institutional review board approval was obtained from the University of Witwatersrand (clearance number M170583) with additional approval for this sub-study from the London School of Hygiene and Tropical Medicine MSc Research and Ethics Committee (reference number 22152). Written informed consent was obtained from individual participants prior to enrolment.

### Data collection and definitions

Demographic and lifestyle data, anthropometric measurements, and blood and urine specimens were collected by locally trained field workers and study nurses. Sex was defined based on participants self-reporting as male or female. Blood and urine samples were stored at 4–8°C until transported to the research laboratory where they were processed and stored at -80°C prior to shipping to the Central Laboratory Services in Johannesburg for testing. For urinary schistosomiasis screening, a 10-milliliter aliquot of urine was filtered through a nucleopore membrane, and the filter examined for eggs by a trained microscopist, using a BS200 Biological Microscope at x10 magnification. Urine samples were examined within 48 hours of receipt of the specimen from the field. A selection of samples underwent a second microscopy read by co-author XGO at the study site for internal quality control and shipped to the National Health Laboratory Services in Johannesburg for external quality control. Serum creatinine was measured using an isotope dilution mass spectrometry (IDMS) traceable Jaffe method. eGFR (ml/min/1.73m ^2^) was calculated using the 2009 Chronic Kidney Disease Epidemiology Collaboration (CKD-EPI) creatinine equation without adjusting for African American ethnicity ^18^. Urine albumin concentration was measured with immunoturbidimetry, and urine creatinine concentration measured with Jaffe’s kinetic method. From these measurements a urine albumin to creatinine ratio (ACR) was calculated (mg/mmol). Kidney dysfunction was defined as a single screen composite outcome of eGFR <60 ml/min/1.73m ^2^ and/or urine ACR >3.0 mg/mmol.

Urinalysis was carried out on fresh urine specimens at the time of collection using Roche Combur 10 urine dipsticks. Urine protein levels were recorded on a scale of 0 to 3+, and urine blood levels on a scale of 0 to 4+. HIV status was either self-reported or based on a rapid antigen test on whole blood. For each participant, the highest level of education and a household assets-based score to assess socioeconomic status were received from the Agincourt HDSS ^19^. Data were collected on water, sanitation, and hygiene (WASH) variables: Household water source, frequency of availability of drinking water and distance to the main household water supply in meters. Freshwater exposure was determined at village level. Each village’s proximity to freshwater was mapped using local cartography ^20^ in conjunction with Google mapping software ^21^. Villages were grouped into three categories based on the distance of their central point to a freshwater source such as a lake or river: <1 kilometer (km), 1 – 2 km and >2 km.

### Statistical analysis

Categorical data are presented as absolute numbers and percentages, and continuous data as means and standard deviations (SD), or medians and interquartile ranges (IQR) depending on their distribution. We conducted a complete case analysis of participants with available data for schistosomiasis, eGFR and ACR. Schistosomiasis infection was recorded as a binary variable; present or absent. Continuous variables eGFR and ACR were recoded as binary: eGFR <60 ml/min/1.73m ^2^ defining a reduced eGFR, and ACR >3.0 mg/mmol defining albuminuria. Kidney dysfunction was subsequently defined as a binary variable – present or absent – based on the composite outcome of an eGFR <60 ml/min/1.73m ^2^ and/or urine ACR >3.0 mg/mmol.

Schistosomiasis point prevalence was calculated as the percentage of the study population with positive urine microscopy for schistosomiasis eggs. Univariable analyses of the crude relationship between covariates and (i) schistosomiasis and (ii) kidney dysfunction were conducted using the chi-squared test.

Age and sex were included as *a priori* variables in the logistic regression model for the association between *S. haematobium* and kidney dysfunction. Additional confounders to be entered into the fully adjusted logistic regression model were determined using a directional acyclic graph ^22^. A likelihood ratio test (LRT) was conducted for the association between *S. haematobium* and kidney dysfunction for the fully adjusted logistic regression model. Clustering of schistosomiasis and kidney dysfunction were controlled for by logistic regression with random effects. The p-value for rho (p<0.001) and values for quadratic approximation (all <0.001) were calculated and provided statistical evidence for the assumption of intra-cluster correlation by village ^23^. Sensitivity analyses were pre-determined to assess for the association of *S. haematobium* with individual indicators of kidney dysfunction (albuminuria, reduced eGFR, haematuria) with odds ratios (OR) calculated using a logistic regression model and the LRT to calculate p-values for these associations. For those excluded from the analyses due to missing data, evidence for a systematic difference between included and excluded participants was assessed using cross-tabulation and the chi-squared test.

Finally, we calculated sensitivity, specificity, and predictive values for the presence of haematuria, detected by urine dipstick, as a screening tool for schistosomiasis when compared to the gold standard diagnostic test of detection of schistosomiasis eggs by urine microscopy. Absolute numbers were entered into a 2 × 2 table, grouping urine dipstick results into true positive (TP), false positive (FP), false negative (FN) and true negative (TN). Results in each cell of the table were then used in the following equations to obtain percentage values: Sensitivity=[TP/(TP+FN)]×100; Specificity=[TN/(TN+FP)]×100; Positive predictive value=[TP/(TP+FP)]×100; Negative predictive value=[TN/(FN+TN)]×100 ^24^. Confidence intervals for sensitivity and specificity were calculated using Clopper-Pearson and the log method used to calculate confidence intervals for positive and negative predictive values ^25^.

All data were captured using electronic case report forms and uploaded onto a secure password-protected online database REDCap ^26^. Data was analysed in Stata version 16 (StataCorp LLC, College Station, TX).

## Results

**Figure 1.**
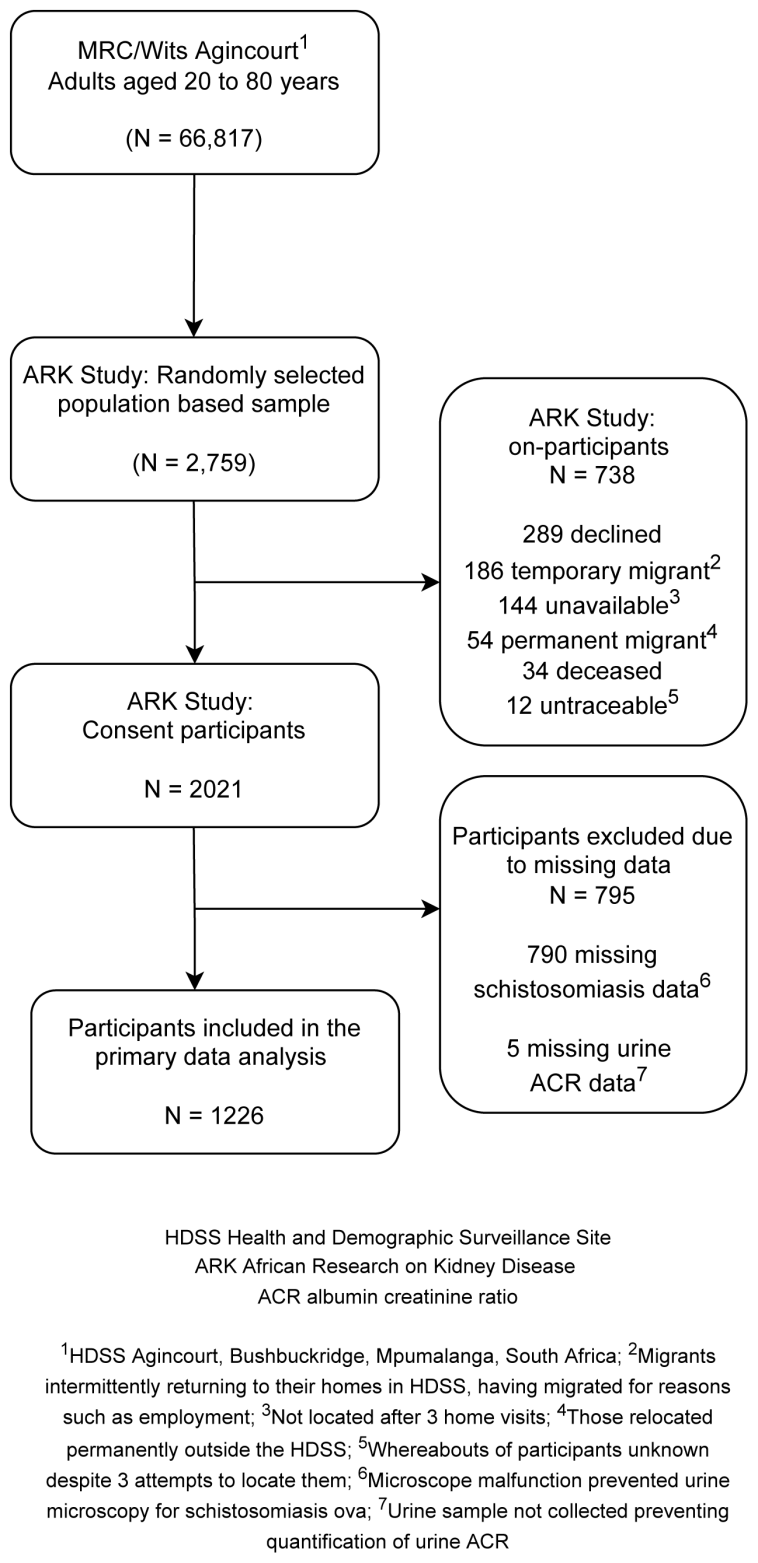
Flow diagram depicting sample selection, participant recruitment, and urinary schistosomiasis screening strategy.

Overall, 2021 of 2759 (73.3%) participants were enrolled into the study. Reasons for non-inclusion are shown in Figure 1. Of those enrolled, 1226 (60.7%) contributed data to this analysis. Remaining participants were excluded due to missing data on schistosomiasis because of microscope malfunction (n=790) and missing data for albuminuria (n=5).

Overall, the median age of participants was 35 years (IQR 27 – 47 years), 58.5% (717/1226) were female, and HIV prevalence was 18.4% (212/1153). The point prevalence of *S. haematobium* infection was 5.1% (63/1226) and risk of *S. haematobium* infection was highest in those aged 20 – 25 years (OR 12.2; 95% CI 4.1 – 36.1) compared to older age groups, and lower in women compared to men (OR 0.51; 95% CI 0.31 – 0.86) ( Table 1). The prevalence of kidney dysfunction was 20.2% (248/1226) and of these: 85.9% (213/248) had moderate albuminuria (ACR 3–30 mg/mmol), 8.5% (21/248) had severe albuminuria (ACR >30 mg/mmol), and 10.9% (27/248) had eGFR < 60 ml/min/1.73m ^2^ ( Table 1). In those with kidney dysfunction, the prevalence of *S. haematobium* was 14.5% compared to 2.8% in those without kidney dysfunction (OR 5.98; 95% CI 3.51– 10.2) and moderate albuminuria was most strongly associated with schistosomiasis infection (OR 7.03; 95% CI 4.09 – 12.1). There was a linear trend with increasing levels of haematuria associated with increasing odds of schistosomiasis (p<0.0001) ( Table 1).

**Table 1.** *Schistosoma haematobium* infection: crude associations by demographic and clinical characteristics.

		Total n (%)	*S. haematobium* absent n (%) (N = 1163)	*S. haematobium* present n (%) (N = 63)	cOR (95% CI)	p-value ^a^
**Sex**	Male	509 (41.5)	473 (40.7)	36 (57.1)	1	0.01
	Female	717 (58.5)	690 (59.3)	27 (42.9)	0.51 (0.31 – 0.86)	
**Age (years)**	20 - 25	259 (21.1)	226 (19.4)	33 (52.4)	12.2 (4.1 – 36.1)	<0.0001
	26 - 30	202 (16.5)	192 (16.5)	10 (15.9)	4.36 (1.34 – 14.2)	
	31 - 35	171 (14.0)	160 (13.8)	11 (17.5)	5.76 (1.78 – 18.6)	
	36 - 40	152 (12.4)	150 (12.9)	2 (3.2)	1.12 (0.20 – 6.17)	
	41 - 45	103 (8.4)	100 (8.6)	3 (4.8)	2.51 (0.55 – 11.5)	
	>45	339 (27.7)	335 (28.8)	4 (6.3)	1	
**Occupation**	Professional	64 (5.4)	62 (5.5)	2 (3.3)	1	0.36
	Clerical/Retail	89 (7.5)	85 (7.6)	4 (6.7)	1.46 (0.26 – 8.27)	
	Labourer/Agriculture	54 (4.6)	51 (4.6)	3 (5.0)	1.82 (0.29 – 11.5)	
	Unskilled/Domestic /	102 (8.6)	828 (73.9)	44 (73.3)	2.31 (0.46 – 11.6)	
	Unemployed	875 (73.9)	94 (8.4)	7 (11.7)	1.65 (0.39 – 6.96)	
**Creatinine (µmol/L)**	Mean (SD)	64 (17)	64 (17)	66 (14)	-	-
**eGFR** **(ml/min/1.73m ^2^)**	<60	27 (2.2)	27 (2.3)	0 (0)	1	0.48 ^b^
	≥60	1199 (97.8)	1136 (97.7)	63 (100)	- ^c^	
**ACR** **(mg/mmol)**	<3	992 (80.9)	965 (83.0)	27 (42.9)	1	<0.0001
	3 - 30	213 (17.4)	178 (15.3)	35 (55.6)	7.03 (4.09 – 12.1)	
	>30	21 (1.7)	20 (1.7)	1 (1.6)	1.79 (0.23 – 13.8)	
**Kidney dysfunction ^d^**	No	978 (79.8)	951 (81.8)	27 (42.9)	1	<0.0001
	Yes	248 (20.2)	212 (18.2)	36 (57.1)	5.98 (3.51 – 10.2)	
**Haematuria**	0	495 (40.4)	793 (68.2)	20 (31.7)	1	<0.0001
	1+	275 (22.4)	142 (12.2)	8 (12.7)	2.23 (0.96 – 5.18)	
	2+	456 (37.2)	85 (7.3)	9 (14.3)	4.20 (1.84 – 9.58)	
	3+	72 (5.9)	64 (5.5)	8 (12.7)	4.96 (2.08 – 11.8)	
	4+	96 (7.8)	78 (6.7)	18 (28.6)	9.15 (4.54 – 18.4)	
**HIV status**	Negative	941 (81.6)	888 (81.3)	53 (86.9)	1	0.27
	Positive	212 (18.4)	204 (18.7)	8 (13.1)	0.66 (0.31 – 1.40)	
**Household water supply**	Tap in house/yard	600 (54.0)	568 (53.8)	32 (57.1)	1	0.50
	Tap in the street	446 (40.1)	424 (40.2)	22 (39.2)	0.92 (0.53 – 1.61)	
	Truck	30 (2.7)	29 (2.7)	1 (1.8)	0.61 (0.08 – 4.65)	
	Other	36 (3.2)	35 (3.3)	1 (1.8)	0.51 (0.07 – 3.83)	
**Household water availability**	Daily	301 (27.1)	284 (26.9)	17 (30.4)	1	0.57
	Irregular	811 (72.9)	772 (73.1)	39 (69.6)	0.84 (0.47 – 1.52)	
**Distance to household** **water supply (meters)**	<50	54 (10.6)	51 (10.5)	3 (13.0)	1	0.86
	50 – 200	192 (37.7)	185 (38.1)	7 (30.4)	0.64 (0.16 – 2.59)	
	>200	263 (51.7)	250 (51.4)	13 (56.5)	0.88 (0.24 – 3.22)	
**Distance to freshwater (kilometers)**	<1	495 (40.4)	469 (40.3)	26 (41.3)	1	0.77
	1 – 2	275 (22.4)	260 (22.4)	15 (23.8)	1.04 (0.54 – 2.00)	
	>2	456 (37.2)	434 (37.3)	22 (34.9)	0.91 (0.51 – 1.64)	

Total with available data: occupation n = 1184; haematuria n=1225; HIV status n=1153; water supply n=1112; water availability n=1112; distance to water supply n=509. All other variables n=1226. cOR crude odds ratio; 95% CI 95% confidence interval; eGFR estimated glomerular filtration rate calculated using the Chronic Kidney Disease-Epidemiology Collaboration equation without African American ethnicity factor; ACR albumin creatinine ratio; HIV human immunodeficiency virus. a calculated from MHodds chi-squared test; b calculated from two sample t-test; c unable to calculate cOR due to data sparsity, d defined as eGFR <60 ml/min/1.73m ^2^ and/or ACR>3 mg/mmol

**Figure 2.**
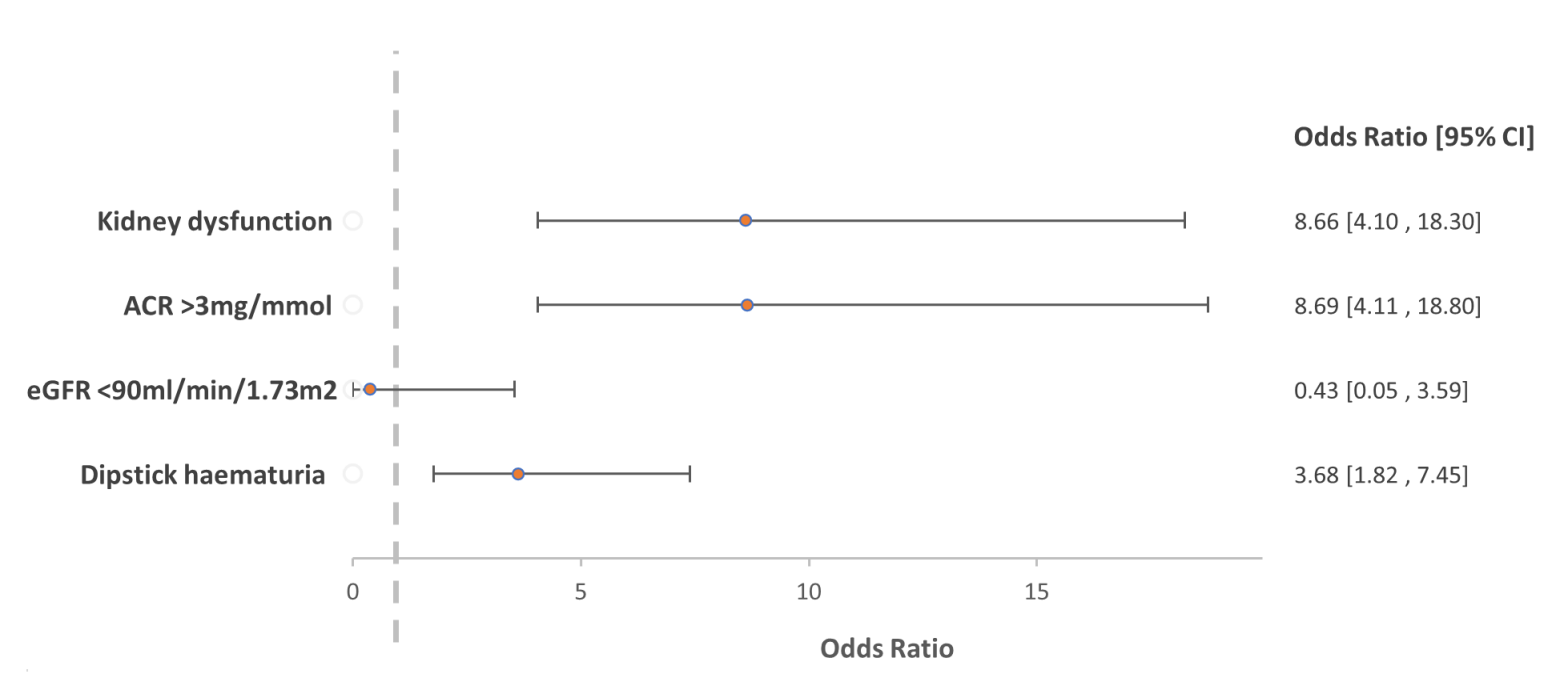
Comparison of the primary logistic regression model for the association with *Schistosoma haematobium* and kidney dysfunction with results from sensitivity analyses of the association of individual markers of kidney dysfunction.

With multivariable analysis ( Figure 2), the adjusted odds ratio for the association between *S. haematobium* infection and kidney dysfunction was 8.66 (95% CI 4.10 – 18.3). Because analysis of eGFR <60 mL/min/1.73m ^2^ as an outcome variable was not possible due to insufficient data, we used eGFR <90 mL/min/1.73m ^2^ with no evidence of association with *S. haematobium* infection (OR 0.43; 95% CI 0.05 – 3.59).

We conducted sensitivity analyses comparing those included in the analysis to those excluded because of missing data **.** There were no differences in respect to age and sex, however there was a difference in the prevalence of kidney dysfunction, which was higher for those included in the analysis (20.2% *versus* 2.3%).

Table 2 shows calculated sensitivity, specificity, and predictive values of haematuria on urine dipstick as a screening tool for *S. haematobium* infection.

**Table 2.** Calculated sensitivity, specificity and predictive values of haematuria detected on urine dipstick as a screening tool for *Schistosoma haematobium* in the study population.

	Urine microscopy positive ^a^, n	Urine microscopy negative ^a^, n	Total N	
**Haematuria dipstick** **positive, n**	43	369	412	**Positive Predictive Value** 10.4% (95% CI 8.8 – 12.3)
**Haematuria dipstick** **negative, n**	20	793	813	**Negative Predictive Value** 97.5% (95% CI 96.5 – 98.3)
**Total N**	63	1162	1225 ^c^	
	**Sensitivity** 68.3% (95% CI 55.3 – 79.4)	**Specificity** 68.2% (95% CI 65.5 – 70.9)		

^a^urine microscopy for eggs of *Schistosoma haematobium* Missing haematuria data in one participant

## Discussion

In this population-based study of adults residing in rural Mpumalanga Province, South Africa, we found that *S. haematobium* remains endemic, with men twice as likely to have evidence of infection than women. Kidney dysfunction was detected in one in five participants, and *S. haematobium* infection was strongly associated with (moderate) albuminuria and urine dipstick hematuria, but not with reduced eGFR.

This is the first study from South Africa to assess the association of endemic *S. haematobium* infection with markers of kidney dysfunction, and one of a few studies from the Mpumalanga province that has performed population screening. There are however limitations, including the absence of screening for those younger than 20 years which is likely to underestimate prevalent infection; exclusion of 795 participants because of missing data with a higher prevalence of kidney dysfunction in those included in the final analysis, reducing generalizability of the study; and the use of a single urine microscopy result to diagnose schistosomiasis compared to the gold standard three separate samples for microscopy ^27^ with a possible underestimate in point prevalence. Any misclassification of schistosomiasis infection is likely to be non-differential which biases effect estimates towards the null. Our results for sensitivity, specificity, PPV and NPV of dipstick haematuria for *S. haematobium* infection should also be viewed in light of this possible underestimation.

In endemic areas, where access to the gold standard diagnostic urine microscopy is unavailable, urine dipstick can be a useful screening tool for *S. haematobium*. The high NPV found in our study supports this approach and is helpful for ruling out those who don’t need investigation or treatment. In the absence of access to additional diagnostic testing, a single dose of praziquantel in those who present with hematuria is recommended in endemic areas in South Africa. One caveat to this recommendation is the need to “exclude glomerulonephritis”, which is impractical given the paucity of specialist nephrology services ^28^. Our estimated prevalence of kidney dysfunction was higher than that of previous studies for sub-Saharan Africa ^18, 29^, almost solely based a higher prevalence of albuminuria. Despite the strong association between schistosomiasis and kidney dysfunction, there is limited prior evidence for a direct causal relationship between *S. haematobium* and intrinsic kidney disease. Future studies are needed to understand causal mechanisms for albuminuria in this group. Aside from *S. haematobium,* other important causes of albuminuria to consider include HIV, tuberculosis and NCDs, of which hypertension is most common and with diabetes on the rise.

The WHO-recommended core strategic intervention of preventative chemotherapy in South Africa aims to eliminate schistosomiasis as a public health problem ^3^. Despite this recommendation, there are no current public health programs targeting schistosomiasis elimination in South Africa. Our calculated prevalence for schistosomiasis in the adult population of 5.1%, alongside a recent prevalence study in both adults and children of more than 30% ^4^, supports the need for targeted public health interventions in the Mpumulanga region. The Neglected Topical Diseases Sustainable Development Goals highlight preventive chemotherapy with praziquantel, access to safer water, sanitation and hygiene, and vector control as public health priorities in the control of schistosomiasis ^1^. Additionally, the detection of haematuria and/or proteinuria on urine dipstick should prompt investigation and individual case management of schistosomiasis infection given the strong associations with *S. haematobium* demonstrated by our study.

## Conclusions

*S. haematobium* infection is endemic in South Africa and strongly associated with hematuria and moderate albuminuria as markers of kidney dysfunction used for diagnosing CKD. Further studies are necessary to investigate causal mechanisms for albuminuria (the most common abnormality) in those with *S. haematobium* infection that will inform future guidelines for management in endemic countries, many of whom are in Africa and likely to remain resource-limited.

## Data Availability

WIReDSpace: Dataset From: Forgotten but not gone in rural South Africa: Urinary schistosomiasis and implications for chronic kidney disease screening in endemic countries, https://doi.org/10.54223/uniwitwatersrand-10539-33712 ^30^ This project contains the following underlying data: Agincourt_schistodata_Excel.xlsx WIReDSpace: Dataset From: Forgotten but not gone in rural South Africa: Urinary schistosomiasis and implications for chronic kidney disease screening in endemic countries, https://doi.org/10.54223/uniwitwatersrand-10539-33712 ^30^ This project contains the following extended data: Phase 1 ARK Study - Agincourt _ REDCap.pdf Schisto_table_figures_submission.docx Dataset_agincourt.numbers Data are available under the terms of the Creative Commons Attribution 4.0 International license (CC-BY 4.0).
