# Supplementary material for: Forgotten but not gone in rural South Africa: Urinary schistosomiasis and implications for chronic kidney disease screening in endemic countries
Source: Wellcome Open Res. 2023 Aug 24;8:68. Originally published 2023 Feb 10. [Version 3] doi: 10.12688/wellcomeopenres.18650.3 (PMC10576187; doi:10.12688/wellcomeopenres.18650.3)
Supplement: Peer review history

# Reviewer report: Reviewer response for version 3

**DOI:** 10.21956/wellcomeopenres.22087.r65812

**Published:** 2023-10-13

**Authors:** Peter Makaula

**Affiliations:** 1 Research for Health Environment and Development, Mangochi, Malawi

I am now satisfied with the revisions the authors have made.

Is the work clearly and accurately presented and does it cite the current literature?

Yes

If applicable, is the statistical analysis and its interpretation appropriate?

Yes

Are all the source data underlying the results available to ensure full reproducibility?

Yes

Is the study design appropriate and is the work technically sound?

Yes

Are the conclusions drawn adequately supported by the results?

Partly

Are sufficient details of methods and analysis provided to allow replication by others?

Yes

Reviewer Expertise:

Neglected tropical diseases, community-based health interventions

I confirm that I have read this submission and believe that I have an appropriate level of expertise to confirm that it is of an acceptable scientific standard.

# Reviewer report: Reviewer response for version 3

**DOI:** 10.21956/wellcomeopenres.22087.r65813

**Published:** 2023-09-19

**Authors:** Olusola Ojurongbe

**Affiliations:** 1 Ladoke Akintola University of Technology, Osogbo, Nigeria

The authors have made a substantial and satisfactory review of the manuscript.

Is the work clearly and accurately presented and does it cite the current literature?

Yes

If applicable, is the statistical analysis and its interpretation appropriate?

Yes

Are all the source data underlying the results available to ensure full reproducibility?

Yes

Is the study design appropriate and is the work technically sound?

Yes

Are the conclusions drawn adequately supported by the results?

Partly

Are sufficient details of methods and analysis provided to allow replication by others?

Yes

Reviewer Expertise:

Medical Parasitology, Genomics of infections diseases

I confirm that I have read this submission and believe that I have an appropriate level of expertise to confirm that it is of an acceptable scientific standard.

# Reviewer report: Reviewer response for version 1

**DOI:** 10.21956/wellcomeopenres.20680.r61846

**Published:** 2023-07-31

**Authors:** Peter Makaula

**Affiliations:** 1 Research for Health Environment and Development, Mangochi, Malawi

Forgotten but not gone in rural South Africa: Urinary schistosomiasis and implications for chronic kidney disease screening in endemic countries

 Craik A, Gondwe M, Mayindi N, Chipungu S, Khoza B, Gómez-Olivé X, Tollman S, Frean J *et al.*

**Please provide a constructive assessment of the study, detailing your thoughts and recommendations for the author. Also, please state if there are any aspects of the study you have not been able to assess.**

 Thank you for the opportunity to review this paper. The authors should be commended for writing this paper on an important topic to validate association of non-communicable diseases and neglected tropical diseases to foster understanding the issues for policy formulation in developing countries. While the authors have done a good job in putting together this paper, there are some areas that will need to be revised to improve the general quality of the manuscript. Below are some comments for their consideration:

1. **Title** - the wording gives a contradictory impression that the study is about rural South Africa, while at the same time referring to other endemic countries. The title and extrapolations should be confined to South Africa considering several limitations of the study also acknowledged by the authors in the Discussion section.
2. **Abstract, Methods** – the second sentence needs to be revised so many “and” used.
3. **Abstract, Results** – indicate percentage of those whose data were available and merge the first two sentences into one.
4. **Abstract, Discussion** – this should be renamed “Discussion and conclusion” or just “Conclusion”.
5. **Introduction** – the authors write “ *The lifecycle of S. haematobium begins with the shedding of eggs from the urine of infected hosts.*” Please specify infected definitive human hosts to differentiate it from intermediate snail hosts mentioned later in the write up.
6. **Methods, Study design and participants** – this subsection needs to be revised. While the subtitle mentions design, “population-based cohort study” have been mentioned only in Abstract – methods and Discussion but no such information is included in this subsection. Later, information unrelated to the subtitle about study area and ethical approvals are included. Consider introducing own subsections for ‘participants selection process’; ‘study area’; ‘ethical considerations’ etc.
7. **Methods, Data collection and definitions** – similarly to the above observation, this subsection has mainly reported on data collection, but no definitions are included as alluded in the subtitle. The definitions are appearing in the next subsection “Statistical analysis”.
8. **Results** - Figure 1, it is not clear what authors mean by “ *ARK Study: on-participants N = 738*” is indicated as excluded in the flow diagram. The reasons for non-inclusion shown in this panel should be first described in Results section before referring the reader to Figure 1. Also consider moving Figure 1 and its associated text to Methods, where the selection process of participants is described.
9. **Results** – the presentation of the Results could benefit from subsections such as “Socio-demographic characteristics of the study participants”; “Association of *Schistosoma haematobium* by demographic and clinical characteristics” etc. to make it easier for a reader to follow.
10. **Discussion** – the limitations of the study should come at the end after findings of the study are discussed (before recommendations for future research.
11. **Conclusion** – authors should consider rewriting “ *S. haematobium infection is endemic [in some parts of] South Africa*” as they reported that it is only mostly found in Limpopo and Mpumalanga provinces.
12. **Conclusion** – authors should highlight key conclusions from the study. Remove the part that reads “ *Further studies are necessary to investigate causal mechanisms for albuminuria (the most common abnormality) in those with S. haematobium infection that will inform future guidelines for management in endemic countries, many of whom are in Africa and likely to remain resource limited*” from to the Discussion section.

Is the work clearly and accurately presented and does it cite the current literature?

Yes

If applicable, is the statistical analysis and its interpretation appropriate?

Yes

Are all the source data underlying the results available to ensure full reproducibility?

Yes

Is the study design appropriate and is the work technically sound?

Yes

Are the conclusions drawn adequately supported by the results?

Partly

Are sufficient details of methods and analysis provided to allow replication by others?

Yes

Reviewer Expertise:

Public health, neglected tropical diseases, noncommunicable diseases

I confirm that I have read this submission and believe that I have an appropriate level of expertise to confirm that it is of an acceptable scientific standard, however I have significant reservations, as outlined above.

# Response

**Published:** 2023-08-14

**Authors:** alison craik

**Affiliations:** London School of Hygiene & Tropical Medicine, UK

Many thanks for your suggestions and comments. Please see our responses below:

 1. The study was conducted within one region of rural South Africa, where we demonstrate a significant burden of infection with schistosomiasis. *Schistosoma haematobium *is endemic in large parts of sub-Saharan Africa and our findings are likely to be relevant to other populations residing in these regions. Our title aims to highlight this. We do not mean to generalise, however we do think it is important to draw attention to the likelihood that populations residing in *Schistsoma haematobium *endemic areas may well have similar findings to those found within our study population in South Africa.

 2. Many thanks for highlighting, the sentence has been re-worded.

 3, 4, 5. Thank-you for the suggestion, the changes have been made.

 6, 7. The subsections have been revised as suggested.

 8. Our apologies for this error. The figure is meant to read "Non-participants" and has been amended. A comment has been added to the text body regarding non-participants however not all reasons have been included in the text due to the large number of different reasons. These can be seen, in full, within the figure.

 9, 10, 11. Thank-you for the suggestion, the changes have been made.

 12. The comment regarding implications for future work has been removed from the conclusion and, instead, added into the discussion section.

# Reviewer report: Reviewer response for version 1

**DOI:** 10.21956/wellcomeopenres.20680.r61833

**Published:** 2023-07-31

**Authors:** Olusola Ojurongbe

**Affiliations:** 1 Ladoke Akintola University of Technology, Osogbo, Nigeria

Craik et al. presented the study of urinary schistosomiasis and its implications for chronic kidney disease. Understanding the implications of urinary schistosomiasis for chronic kidney disease is crucial, and the presented work provides valuable data and results for further analysis. The study seems to be well-designed and presented, but the authors will need to clarify these observations. 

1. Excluding a whole lot of 790 samples because of the microscope malfunction is not a welcome development. This large chunk of data would have significantly contributed to the study outcome. Why can't the authors preserve these samples and re-analyze them? 
2. The author should clarify this statement " *Because analysis of eGFR <60 mL/min/1.73m2 as an outcome variable was not possible due to insufficient data*". Was the eGFR not conducted on the 790 individuals without schistosomiasis results due to microscope malfunction? 
3. In Table 2 table the author presented the Creatinine (μmol/L) as Mean (SD), whereas the heading of the table says n (%). This is a bit confusing. The author should correct it appropriately. 
4. The author listed several limitations in the study, including using single urine microscopy to diagnose schistosomiasis, which I believe has a significant impact on the outcome of this study. In addition, the inability of the authors to administer PZQ to schistosomiasis-positive participants and analyze their post-treatment sample is an additional limitation. This would have helped to see if the observed CKD in those positive was due to schistosomiasis or other factors not investigated. The authors should comment on this. 

Is the work clearly and accurately presented and does it cite the current literature?

Yes

If applicable, is the statistical analysis and its interpretation appropriate?

Yes

Are all the source data underlying the results available to ensure full reproducibility?

Yes

Is the study design appropriate and is the work technically sound?

Yes

Are the conclusions drawn adequately supported by the results?

Partly

Are sufficient details of methods and analysis provided to allow replication by others?

Yes

Reviewer Expertise:

Medical Parasitology

I confirm that I have read this submission and believe that I have an appropriate level of expertise to confirm that it is of an acceptable scientific standard, however I have significant reservations, as outlined above.

# Response

**Published:** 2023-08-11

**Authors:** alison craik

**Affiliations:** London School of Hygiene & Tropical Medicine, UK

Many thanks for your comments and suggestions. Please find our responses to your comments below:

 1. We recognise that the missing data for schistosomiasis is a limitation of the study, and that the generalizability of the study is reduced due to the systematic differences in markers of kidney dysfunction when comparing those participant groups with and without available data for schistosomiasis. Urine microscopy for the ova of schistosomiasis can only be conducted on fresh urine specimens and not those that have been frozen.

 2. This statement is referring to insufficient data rather than missing data. Very few participants in our study had a reduction in eGFR to any degree. Furthermore the number of those with an eGFR of less than 60 was so small that it did not allow for the multivariable analysis which we had originally planned for. The multivariable analysis was therefore conducted using those with any degree in reduction of eGFR as a variable.

 3. Many thanks for highlighting this. This row has been removed from the table and the mean creatinine value added into the text body of the results section.

 4. We agree that the use of single urine microscopy for the diagnosis of urinary schistosomiasis is a limitation of the study. Microscopy for schistosomiasis ova is sepcific but not sensitive, and the number of people infected with schistosomiasis in our study is likely to be an under-estimate. The design of the study was an observational cohort study and not an interventional study which therefore did not allow for us to measure the effects of an intervention such as praziquantel. This, however, is an important research question going forward and future research is needed to evaluate whether the albuminuria associated with schistosomiasis is transient or persistent after treatment with praziquantel.
